# Fatal invasive *Aspergillus* infection in an elderly patient with hepatitis E: A case report and literature review

**DOI:** 10.1097/MD.0000000000040103

**Published:** 2024-10-25

**Authors:** Junjun Wu, Jin Yu, Huaming Li, Yufang Wang, Rong Xu

**Affiliations:** a Department of Gastroenterology, Hangzhou Third People’s Hospital, Hangzhou, China.

**Keywords:** *Aspergillus*, hepatic failure, hepatitis E virus, invasive fungal infection

## Abstract

**Rationale:**

Elderly patients with acute liver failure are highly susceptible to severe complications, such as invasive fungal infections, due to weakened immune systems and altered gut microbiota. A thorough understanding of liver failure and opportunistic infections is crucial for effective management.

**Patient concerns:**

An 84-year-old male with acute liver failure from hepatitis E experienced worsening jaundice despite standard treatments. He also developed respiratory symptoms, including blood-streaked sputum, raising concerns about a potential fungal infection.

**Diagnoses:**

The patient was diagnosed with acute liver failure secondary to hepatitis E and an invasive fungal infection caused by Aspergillus fumigatus. Initial treatments included artificial liver plasma exchange and antifungal prophylaxis. Further diagnostics, including bronchoscopy and next-generation sequencing of alveolar lavage fluid, confirmed the Aspergillus infection.

**Lessons:**

Elderly liver failure patients are particularly prone to opportunistic infections, underscoring the need for vigilant monitoring and early intervention. Despite aggressive treatments, including antifungal therapy and artificial liver support, prognosis remains poor, highlighting the importance of prompt diagnosis and comprehensive management to enhance patient outcomes.

## 1. Introduction

Liver failure can be triggered by viruses, drug use, alcohol, or other factors inducing serious injury to liver tissues and liver cell necrosis. This causes severe liver function impairment and even decompensation, eventually leading to hyperjaundice, coagulation dysfunction, ascites, hepatic encephalopathy, and a series of clinical manifestations of the syndrome.^[1]^ Cirrhosis is the end stage of diffuse liver damage caused by long-term or repeated exposure to one or more causative factors. In the early stages, due to the strong compensatory function of the liver, no obvious symptoms are observed. As the disease progresses, the condition can advance to the decompensation stage. Common complications include gastrointestinal bleeding, hepatic encephalopathy, secondary infection, hypersplenism, ascites, and other complications. Multiple organ dysfunctions such as hepatorenal syndrome and hepatopulmonary syndrome can also occur.^[2]^ Generally, approximately 10.6 million patients with cirrhosis are hospitalized each year, of which 1.32 million cases are fatal, placing a significant burden on the global economy.^[3]^ In patients with liver failure, immunosuppression, intestinal flora disturbance, repeated hospitalization, malnutrition, mucosal barrier destruction, and long-term use of broad-spectrum antibiotics, among other factors, can cause secondary infections. These typically include spontaneous peritonitis, pneumonia, and other bacterial infections, with fungal infections being one of the most serious complications that require attention.

Fungi, ubiquitous in nature, serve as opportunistic pathogens affecting billions of patients worldwide, According to the latest estimates, the annual incidence of invasive fungal infections is 6.5 million, and the number of deaths is 3.8 million, of which 2.5 million people die directly due to invasive fungal infections.^[4]^ The fatality rate in most fungal infections is as high as 30%, reaching 50% in patients with fungal peritonitis.^[5]^ Therefore, improving our understanding of fungal pathogenesis and achieving early diagnosis are crucial to improving cure rates. Although early diagnosis and treatment improves the prognosis, this is challenging in critically ill, non-neutropenic patients. These individuals often lack clinical symptoms, display atypical radiological signs, and have limited information from fungal cultures, resulting in delayed treatment. Patients with advanced or acute liver disease are increasingly susceptible to bacterial and fungal infections, due to immune impairment,^[6,7]^ increased intestinal permeability, frequent use of corticosteroids, malnutrition, and invasive surgeries. Herein, we analyze a case of secondary fungal infection in a patient with acute hepatitis E–induced liver failure and review the literature on secondary fungal infection in end-stage liver disease. Our goal is to improve our understanding of fungal infection, raise awareness of clinical symptoms in at-risk patients, and, ultimately, facilitate timely diagnosis and early treatment. This case was approved by the Ethics Committee of Hangzhou Third People’s Hospital (ethics number: 2024KA074).

## 2. Case presentation

**History of present illness:** Three days before hospitalization, an 84-year-old male had a yellow urine color with no obvious cause (wherein the color gradually deepened without any decreased urine volume), no obvious abdominal pain and distension, no nausea, vomiting, no skin pruritus, no skin petechial ecchymosis, no gingival bleeding, epistaxis, and no cold or fever. The patient was not concerned at that time and did not seek medical attention. One day before hospitalization, he experienced yellow watery stools 8 times and bloody stool 1 time, without any apparent cause. The volume was moderate, and there was no postural tachycardia. This issue improved on its own, and the patient did not seek medical attention. Ten hours before hospital visit, his family found that he had slurred speech, salivation, yellow skin, and eyes; felt weak; and had a poor appetite. He showed no limb twitching, no adverse limb movements, no confusion, and no headache or dizziness, and he was rushed to the emergency department of our hospital. Initial tests showed glutamic alanine transaminase 2471 U/L, glutamic oxaloacetic transaminase 2228 U/L, glucose 2.90 mmol/L, lactate dehydrogenase 529 U/L, blood ammonia 100 μmol/L, coagulation function tips: Activated partial thromboplastin time 51.7 seconds, D-dimer 4.56 mg/L, fibrinogen 1.11 g/L, international normalized ratio 2.69, prothrombin time 28.7 seconds, thrombin time 28.9 seconds, prothrombin activity 12%. B-ultrasonography indicated changes in liver echo, significant thickening of the gallbladder wall, multiple gallbladder stones, enlarged spleen, and abdominal fluid accumulation, leading to a diagnosis of liver insufficiency and abdominal infection. After treatment with cefotaxime sodium injection, reduced glutathione injection, and fluid rehydration, the patient’s speech became clear, no salivation was found, peripheral blood glucose was remeasured at 4.2 mmol/L. The patient remained in the hospital for further diagnosis and treatment.

**Personal history:** History of hypertension, family history, but no history of hereditary or similar diseases.

**Physical examination:** Temperature 36°C, pulse rate: 93 beats/min, respiration rate: 20 breaths/min, blood pressure: 126/67 mm Hg, clear mind, tongue extension in the center, obvious yellow staining in the skin and sclera, no spider nevi or liver palms, asterixis did not lead out, respiratory sounds in both lungs were clear, no dry and wet rales heard, no abdominal varicose veins observed, gastrointestinal type and peristaltic wave were not observed, soft touch, slight tenderness in the right upper abdomen, no reflex pain or muscle tension. Liver and spleen could not be reached below the ribs, Murphy’s sign was suspected positive, shifting dullness was positive, bowel sounds was 3 times/min, both lower limbs were not swollen. Babinski sign (−).

**Figure 1.**
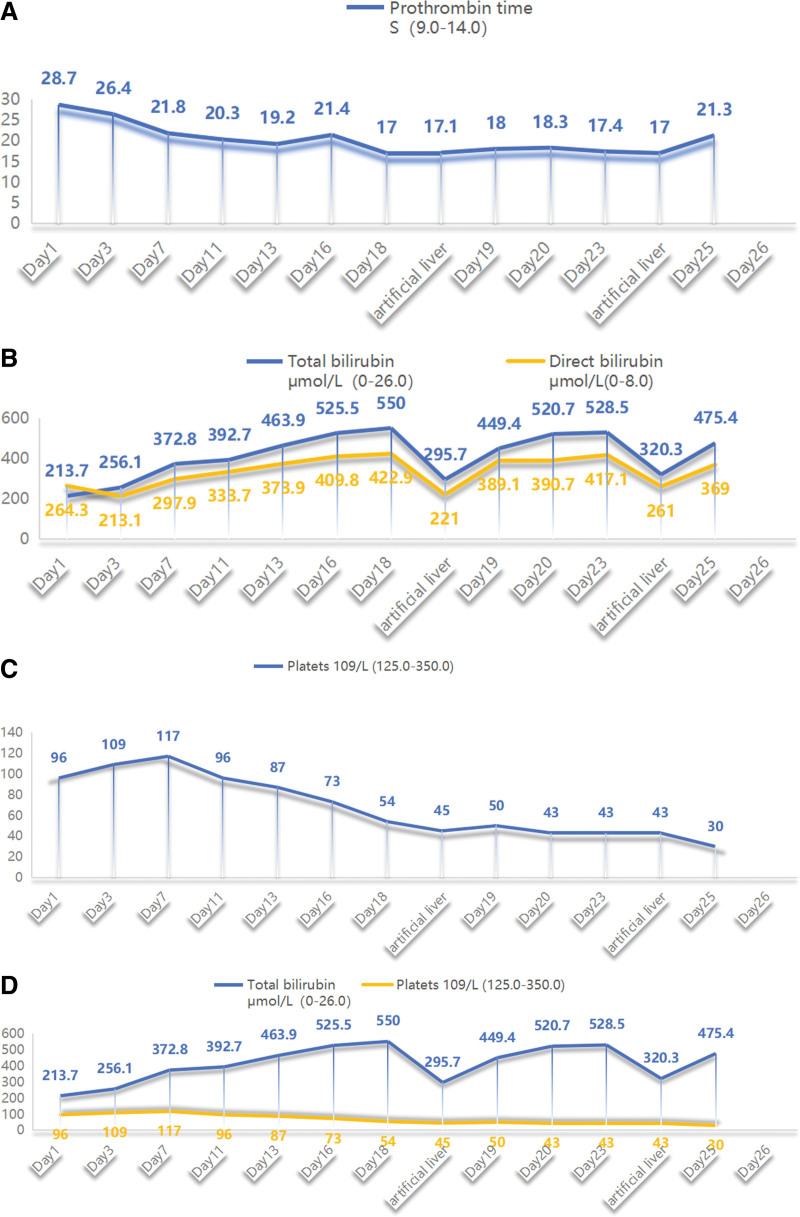
Illustrates the trends of prothrombin time (PT), bilirubin levels, and platelet counts before and after treatment.

**Figure 2.**
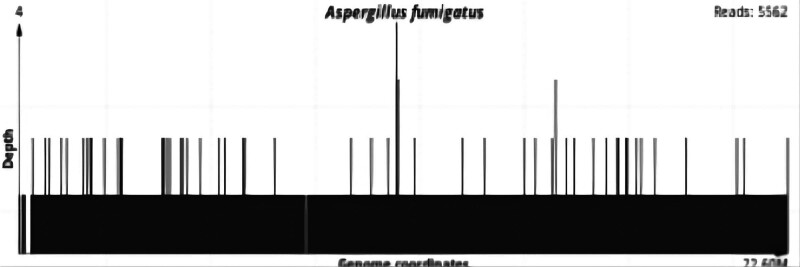
Illustrates the sequence map of Aspergillus fumigatus cultured using next-generation sequencing (NGS).

**Laboratory examination:** The first day before admission, the following findings were recorded: coagulation function: activated partial thromboplastin time (APTT) 51.7 seconds, D-dimer 4.56 mg/L, fibrinogen 1.11 g/L, international normalized ratio 2.69, prothrombin time (PT) 28.7 seconds. Blood ammonia: 70 μmol/L. Blood routine and C-reactive protein: 14.1 mg/L, white blood cells 4.8 × 10^9^/L, hemoglobin 115 g/L, platelets 117 × 10^9^/L, neutrophil 86.0%; reticulocyte count% at 1.0%; procalcitonin 0.454 ng/mL, B-type natriuretic peptide precursor 1179.8 pg/mL. Biochemical: albumin 27.4 g/L, alanine aminotransferase 537 U/L, aspartate aminotransferase 154 U/L, urea 2.68 mmol/L, creatinine 53 μmol/L, direct bilirubin 297.9 μmol/L, fructosamine 3.13 mmol/L, glucose 5.89 mmol/L, indirect bilirubin 74.9 μmol/L, potassium 3.44 mmol/L, lactate dehydrogenase 272 U/L, total bilirubin 372.8 μmol/L, total cholesterol 2.42 mmol/L, triglyceride 1.01 mmol/L, free fatty acid 1290 μmol/L. Coagulation and D-dimer: APTT 62.4 seconds, D-dimer 3.40 mg/L, fibrinogen 0.72 g/L, international normalized ratio 1.96, PT 21.8 seconds. Hepatitis B panel: Hepatitis B core antibody positive, remaining negative; Hepatitis E IgM antibody positive; hepatitis A, B, and C antibodies negative. On the 24th day: albumin 28.6 g/L, alanine aminotransferase 77 U/L, aspartate aminotransferase 73 U/L, urea 13.09 mmol/L, calcium 1.99 mmol/L, chlorine 91 mmol/L, creatinine 68 μmol/L, direct bilirubin 330.8 μmol/L, fructosamine 2.18 mmol/L, glutamine acyltransferase 25 U/L, glucose 7.72 mmol/L, indirect bilirubin 101.9 μmol/L, potassium 4.85 mmol/L, total bilirubin 432.7 μmol/L. On the 25th day: Blood routine and C-reactive protein: 34.2 mg/L, white blood cells 16.1 × 10^9^/L, hemoglobin 103 g/L, platelets 31 × 10^9^/L, neutrophil percentage 88.3%. Coagulation and D-dimer: APTT 81.5 seconds, D-dimer 2.51 mg/L, fibrinogen 1.01 g/L, international normalized ratio 1.91, PT 21.3 seconds (Table 1; Fig. 1). Alveolar lavage fluid NGS: *Aspergillus fumigatus* (Fig. 2), *Acetobacter* smear negative.

**Table 1:** Presents the laboratory data of the patient during his treatment.

Laboratory paremeters	Day 1	Day 3	Day 7	Day 11	Day 13	Day 16	Day 18	Artificial liver	Day 19	Day 20	Day 23	Artificial liver	Day 25	Day 26
ALT U/L (0–50)	2471	1424	537	239	175	127	109		71	92	109	61	90	Die
AST U/L (0–50)	2228	807	157	66	71	65	63		54	71	75	38	82	
ALP U/L (45.0–125.0)		159	113	108	103	95	82		60	64			54	
Total bilirubin μmol/L (0–26.0)	213.7	256.1	372.8	392.7	463.9	525.5	550	295.7	449.4	520.7	528.5	320.3	475.4	
Direct bilirubin μmol/L (0–8.0)	264.3	213.1	297.9	333.7	373.9	409.8	422.9	221	389.1	390.7	417.1	261	369	
Total protein g/L (65.0–85.0)		56.1	55.9	53.4	57.5	57.5	55		48.5	49.1	54.8		41.3	
Albumin g/L (40.0–55.0)		28.1	27.4	26.8	30.2	32.3	32		32.2	31.9	29.8		26	
Prothrombin time S (9.0–14.0)	28.7	26.4	21.8	20.3	19.2	21.4	17	17.1	18	18.3	17.4	17	21.3	
INR (0.8–1.24)	2.69	2.38	1.96	1.82	1.72	1.92	1.55	1.56	1.61	4.64	1.59	1.56	1.91	
PPA %	27.6	31.1	41.7	47.0	51.8	43.1	65.1	64.3	58.3	56.5	62.2	65.1	43.4	
WBC 10^9^/L (3.5–9.5)	8.8	6.5	4.8	7.8	7.9	9.1	7.7	10.4	9.7	8.3		16.7	16.1	
N 10^9^/L (3.5–9.5)	6.48	4.02	4.13	6.42	6.91	7.06	6.8	9.13	7.83	7.14		14.4	14.2	
PLT 10^9^/L (125.0–350.0)	96	109	117	96	87	73	54	45	50	43	43	43	30	
CRPmg/L (0.0–10.0)	18.4	15.2	14.1	6.8	7.6	3.7		1.6	1	3.6		3	17.2	

ALP = alkaline phosphatase, ALT = glutamic-pyruvic transaminase; AST = glutamic oxalacetic transaminase, CRP = C-reactive protein, INR = International Normalized Ratio, N = neutrophil absolute value, PLT = platets, WBC = white blood cell.

**Figure 3.**
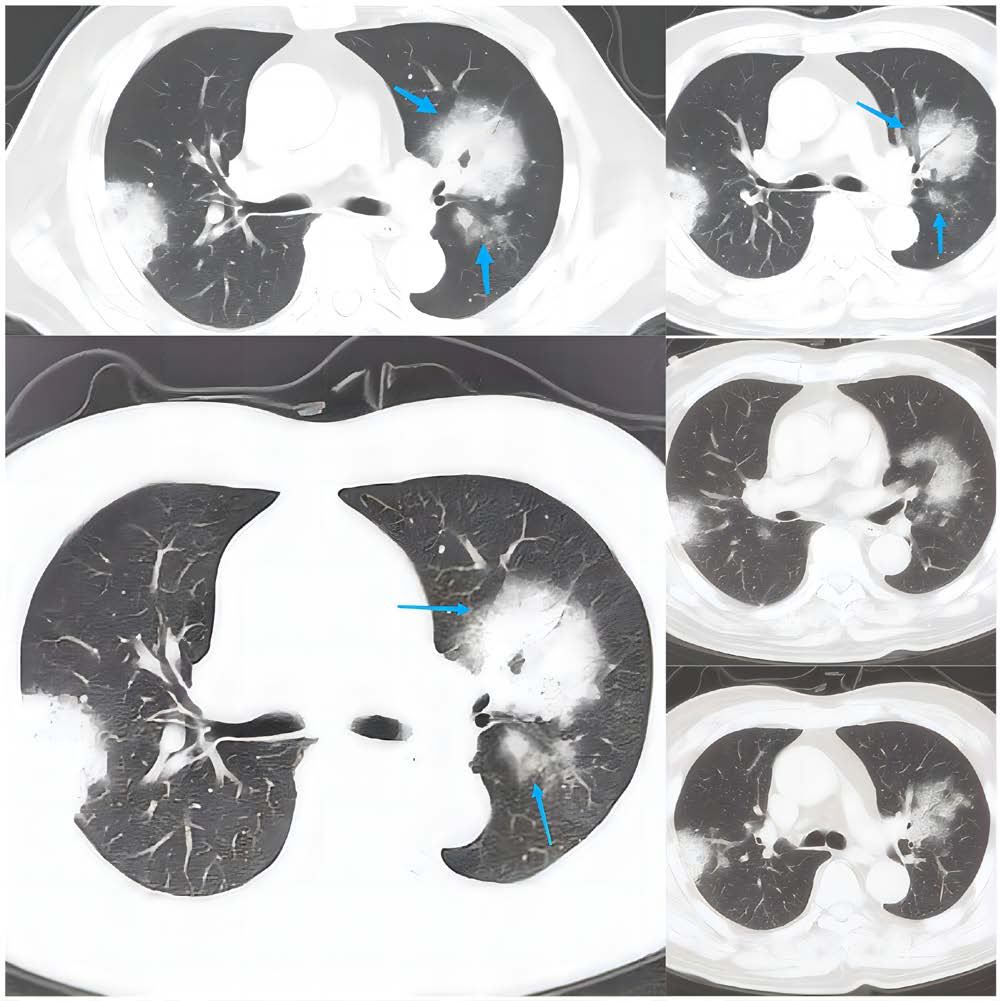
Displays the patient’s complete lung CT scan. The presence of chest tightness and shortness of breath indicated potential infectious lesions in both lungs, with faint shadows observed around the left lung, as indicated by the arrows.

**Figure 4.**
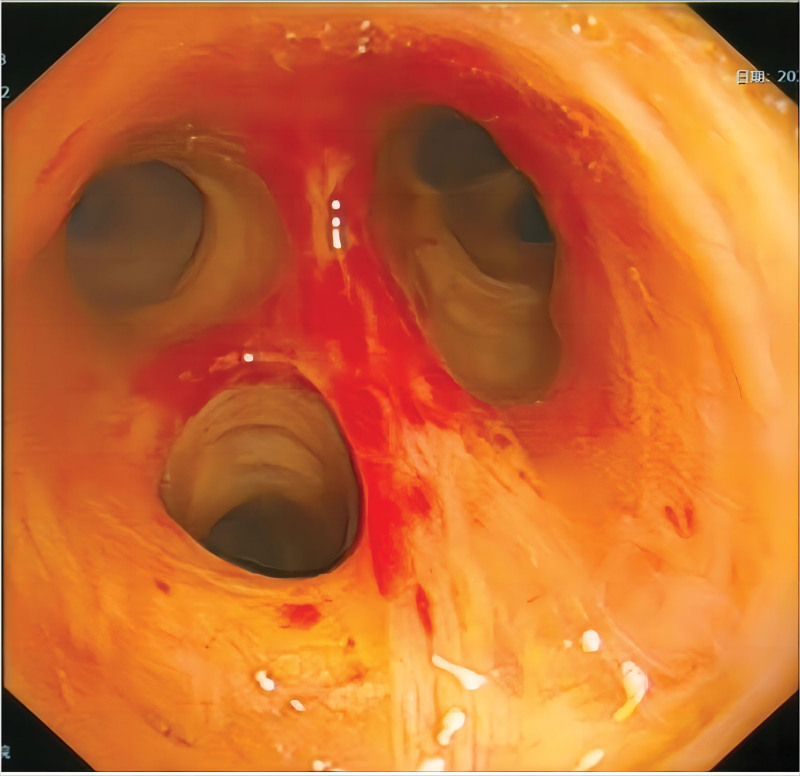
The patient underwent bronchoscopy while experiencing chest tightness and shortness of breath, during which fresh blood was observed in the left upper bronchial lumen.

**Imaging examination:** On admission, a computed tomography (CT) scan of the lungs indicated a low level of inflammation in both lungs with limited pleural effusion on both sides. Multiple small nodules were observed in both lungs; therefore, an annual review was recommended. The abdominal ultrasound indicated the presence of fatty liver and echogenic changes, liver function examination was recommended. The gallbladder wall was clearly thickened and rough. Further interrogation of the patient with clinical and laboratory examination was recommended. Additionally, multiple stones in the gallbladder, splenomegaly, and fluid accumulation in the abdomen were observed. Whole abdominal contrast-enhanced CT indicated the presence of a hepatic cyst, multiple gallstones, and cholecystitis. The local wall of the ascending colon was rough, and the patient was referred for clinical evaluation. In addition, abdominal dropsy, enlargement of the prostate, and calcification of the abdominal aorta were observed. The liver was small in size, with a circular low-density shadow and clear boundary. The intrahepatic bile duct was not dilated. The structure of the hepatic portal was clear. The wall of the gallbladder was rough, with multiple spots and dense shadows inside, and liquid density shadows around the gallbladder fossa. A head magnetic resonance imaging scan showed scattered lacunar infarcts in the basal ganglia on both sides. Pulmonary CT examination after 22 days of treatment indicated infectious lesions in both upper lungs (Fig. 3). Bronchoscopy indicates fresh blood in the left upper bronchial tube lumen (Fig. 4).

### 2.1. Final diagnosis

Acute liver failure, acute severe hepatitis E, hepatorenal syndrome, acute respiratory distress syndrome, metabolic acidosis, and pulmonary *Aspergillus* infection.

**Figure 5.**
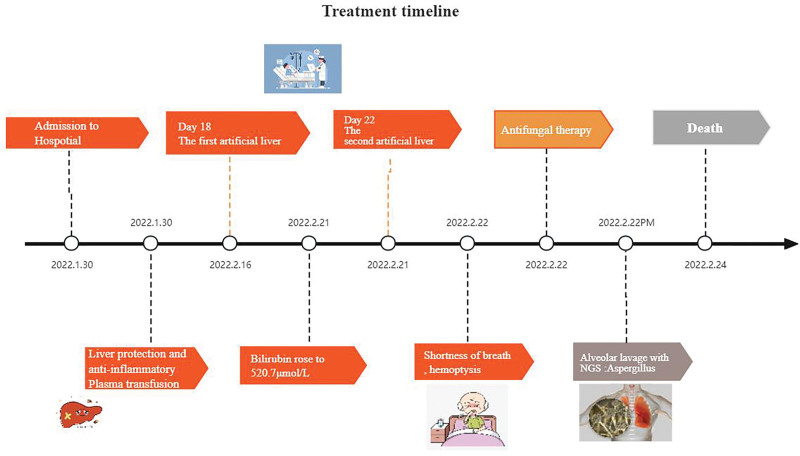
The complete diagnosis and treatment process for the patient.

**Treatment:** Upon admission to hospital, antibiotic (meropenem, 1.0 g, q8h, i.v.) treatment; 150 mg of magnesium isoglycyrrhizinate injection (1 per day) to protect the liver; demetionine butanedisulfonate for injection 1.0 QD to detoxify liver; 8 g of acetylcysteine injection QD to promote liver detoxification; 10 µg of alprostadil micro-pump QD to improve liver microcirculation, to inhibit acid production, protect the stomach, alleviate constipation, and regulate intestinal flora. Additionally, the patient received fluid and sugar supplements and other symptomatic treatment including multiple transfusions of fresh plasma. Despite monitoring liver function and coagulation function, the patient’s condition deteriorated, with total bilirubin increasing to 520.7 μmol/L direct bilirubin to 390.7 μmol/L, indirect bilirubin to 130.0 μmol/L, thromboplastin APTT to 56.0 seconds, D-dimer to 1.66 mg/L; fibrinogen decreasing to 0.89 g/L; international standardized ratio increasing to 1.64; PT increasing to 18.3 seconds with prothrombin activity at 25%. Consequently, after 18 days of treatment, the patient underwent artificial liver treatment twice in the intensive care unit (ICU). On the second day of artificial liver treatment, the patient developed cough and blood-tinged sputum. Reexamination of the lung CT revealed infectious lesions in both lungs, with a faint shadow around the left lung. Bronchoscopy and alveolar lavage fluid examination indicated fungal infection. The following day, caspofungin 50 mg QD was initiated, and 2 days later, alveolar lavage fluid culture results confirmed the presence of *Aspergillus*. Unfortunately, at this stage, the patient experienced sudden chest tightness, shortness of breath, rapid heartbeat, and respiratory arrest. Despite attempts at resuscitation with active cardiac treatment, the patient succumbed to their condition. We used Figure 5 to illustrate the patient’s condition before and after the diagnosis and treatment.

## 3. Discussion

The geriatric patient, with a history of hypertension, presented with clinical symptoms of cholestasis including yellowing of the skin and urine upon admission to the hospital. Despite initial treatment with active liver protection and plasma transfusion, the patient’s condition did not improve, prompting initiation of artificial liver treatment. However, two days after treatment initiation, the patient developed symptoms such as chest tightness, shortness of breath, and hemoptysis. This summary of the treatment process aims to provide insights for managing similar cases in the future.

### 3.1. Hepatic failure

Acute liver failure refers to a series of clinical manifestations such as hyperjaundice, coagulation dysfunction, ascites and hepatic encephalopathy caused by severe liver tissue damage and even decompensation due to alcohol, drug use or viral infection.^[1]^ In addition, patients have low immune function and are susceptible to secondary infection for the following reasons: pathological changes in patients with liver failure include the necrosis of large hepatocytes, leading to a decrease or lack of synthetic complement and antibody, and a decrease in serum opsonization. At the same time, the number and function of Kupffer cells in the liver of patients with advanced liver disease are reduced, making them vulnerable targets for various pathogens; patients with end-stage liver disease may have chronic systemic inflammation due to intestinal flora disorders, the loss of intestinal mucosal integrity and intestinal flora translocation. The liver releases damaged molecular patterns and inflammatory mediators, causing systemic inflammation and multiple organ dysfunction and/or failure, resulting in acute damage to systemic circulatory functions and organ hypoperfusion. This feature is called immunopathology.^[8,9]^ In end-stage liver disease, the physiological mechanism is similar to that of sepsis, both of which occur under the condition of intense systemic inflammation and oxidative stress, involving immune deficiency and continuous activation of immune cells. It is accompanied by an increase in pro-inflammatory cytokines and systemic inflammatory response syndrome.^[10]^ The occurrence of organ failure in the course of the disease is closely related to the prognosis, and it is easy to identify bacterial infection during the disease diagnosis. In the early stage of the disease, due to the impaired function of immune cells and their depletion by apoptosis, the primary infection can worsen or a new secondary infection may develop.^[11–13]^

### 3.2. Invasive aspergillosis

**Figure 6.**
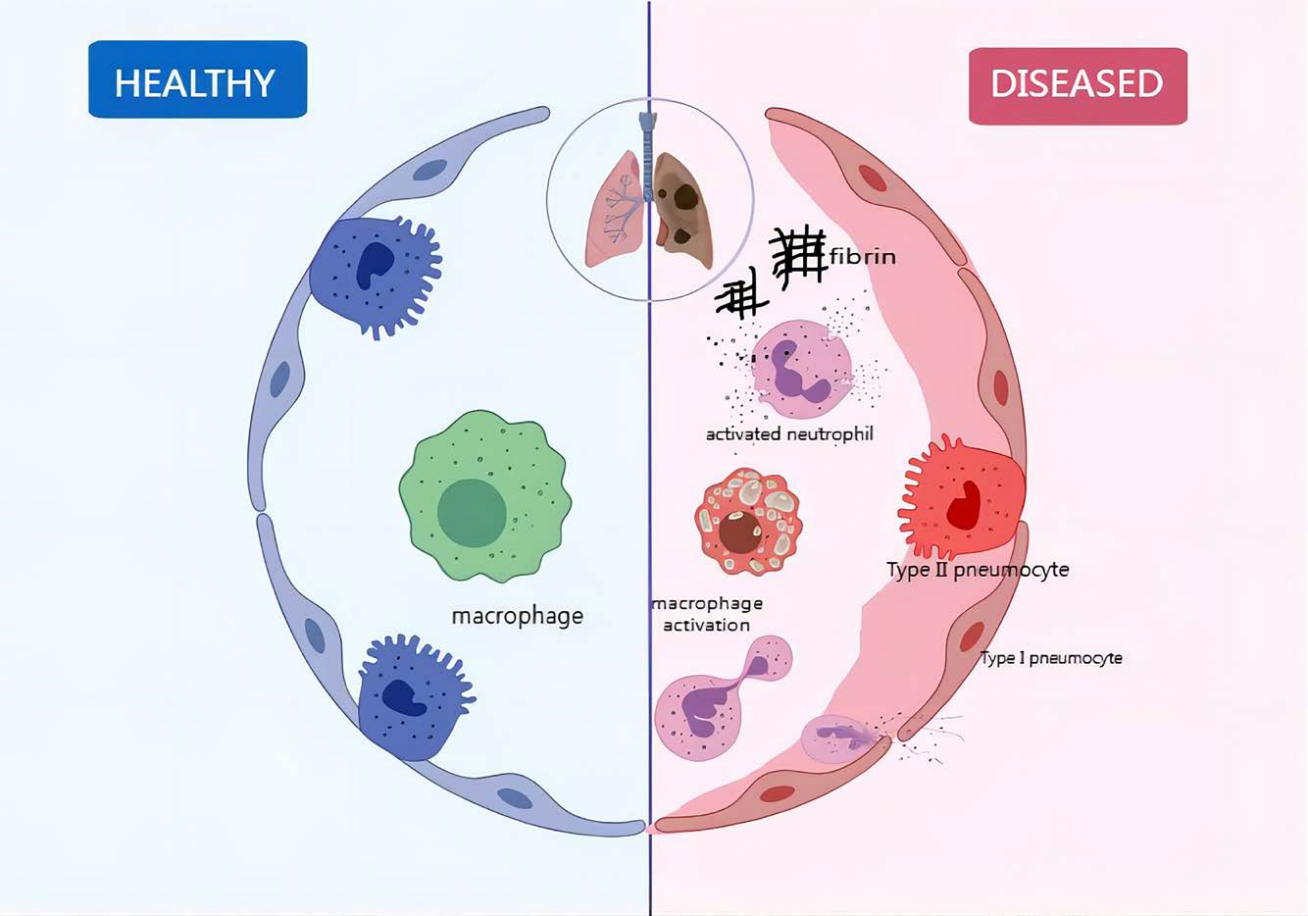
During an infection, alveoli produce a significant amount of pro-inflammatory cytokines, which trigger an inflammatory storm. This response leads to the structural destruction of the alveoli, immune disorders, and other complications, ultimately resulting in patients developing acute respiratory distress syndrome.

Invasive aspergillosis (IA), a major cause of death and morbidity in immunocompromised patients, is often found in patients with long-term neutropenia and/or hematopoietic stem cell transplantation. Concurrently, IA detected during autopsy is often a missed diagnosis.^[14,15]^ As awareness of IA has increased, the rate of detection has increased in patients with chronic obstructive pulmonary disease or cirrhosis or those on long-term steroid therapy.^[16–18]^ IA is a common undiagnosed complication in patients with acute liver failure or end-stage liver disease, with a mortality rate of more than 70%.^[19]^ Platelets have been found to have a growth-inhibiting effect on *Aspergillus* spp. in vitro.^[20]^ Combined with this case, the patient’s platelets were progressively decreased during treatment, which is detrimental to the immune system’s efforts to control the disease, contributing to a poor prognosis. Admission to the ICU is also an independent risk factor for IA infection, as it increases the risk of infection.^[21]^ The patient in this case demonstrated sudden-onset dyspnea and hemoptysis after being admitted to the ICU for the second artificial liver treatment. These symptoms can be related to a large number of pro-inflammatory cytokines being produced in the alveoli during infection, forming an inflammatory storm. This results in structural destruction of the alveoli, immune disorders, and other comprehensive factors, causing acute respiratory distress syndrome in the patient^[21]^ (Fig. 6).

In non-neutropenic diseases, the clinical symptoms of IA are atypical and the diagnostic sensitivity of culture of *Aspergillus* from respiratory secretions is low, Meersseman et al found that only 60% of histologically confirmed cases of pulmonary aspergillosis were positive in bronchoalveolar lavage culture.^[22]^ Moreover, pulmonary CT does not reveal characteristic abnormalities such as cavitation, air crescent sign, or halo sign, limiting its utility. However, in this case, the patient had no clinical manifestations such as cough, phlegm, chest tightness, and shortness of breath in the early stage of his admission. However, pulmonary CT examination revealed changes in pathology, and the diagnosis of *Aspergillus* infection became clear when combined with the results of the alveolar lavage culture. Our findings remind us to be vigilant concerning the risk of fungal infection in similar cases in the future.

Imaging can help us identify the presence of fungal infection; however, the specific causative pathogen is usually not clear. Laboratory examination should be performed to further confirmation of the diagnosis. The galactomannan (GM) detection test is commonly used for clinical diagnosis of fungal infection on serum or alveolar lavage samples.^[23]^ The additional detection of 1, 3-β-D-glucan (BDG) has been used to improve the diagnostic rate for pulmonary *Aspergillus* infection associated with COVID-19.^[24,25]^ Serum analyses for *Aspergillus* IgG antibodies, especially through the lateral flow assay, can play a central role in detecting chronic *Aspergillus* infection and are simpler and faster than other methods.^[26]^ Findings from the spore germination to invasive aspergillosis proliferation phase and its metabolites suggest a connection between the types of acetyl fusarinine C (triacetylfusarinine C), the secretion of TafC, triacetylfusarinine B (TafB), and ferritin ferricarrier. Analysis of these components in urine can help differentiate between invasive pulmonary aspergillosis disease and asymptomatic colonization. The detection sensitivity is 92.3%, and the specificity is 100%, which is superior to galactomannan (GM) and beta-D-glucan (BDG).^[27]^ In view of the limitations of traditional detection methods, metagenomic next-generation sequencing (mNGS) can overcome these challenges, and this method has a high degree of specificity.^[28]^ In our case, mNGS of alveolar lavage fluid also indicated *Aspergillus fumigatus* infection, suggesting that mNGS could be attempted where there are new developments in clinical disease or challenges in making a diagnosis (Fig. 6).

### 3.3. Hepatitis E virus

Hepatitis E virus (HEV) is a 7.2-kb, long, positive RNA virus divided into eight genotypes (HEV-1 to HEV-8), among which HEV-1, HEV-2, HEV-3, HEV-4, and HEV-7 have been confirmed to infect humans.^[29,30]^ The virus is also one of the common causes of viral hepatitis worldwide. Although HEV infection can be self-limiting, it is not uncommon for HEV infection to induce liver parenchymal injury, acute liver failure, and even death, especially in chronic liver diseases such as alcoholic cirrhosis^[31]^ and chronic viral hepatitis B.^[32]^ HEV infection in the third trimester of pregnancy has a high mortality rate, and acute liver failure can occur.^[33,34]^ Elderly people are at risk of HEV coinfection alongside hepatitis B cirrhosis,^[35]^ and it may have increased long-term mortality.^[33,35]^ No correlation has been reported between the viral load of HEV and acute hepatitis E. At the cytokine level, a sharp decline has been observed in IFN-γ levels when progressing from acute hepatitis E to acute hepatitis E failure.^[36]^ Two mutations, namely A317T and V1120I, have been found to significantly increase HEV-1 replication, which may explain the rapid propagation of the virus and the severity of the disease.^[37]^

In summary, the elderly, male patient in the present case study, who was diagnosed with acute hepatitis E failure, was treated with steroids and received artificial liver treatment twice in the ICU. He developed secondary invasive *Aspergillus* infection, which rapidly progressed. Therefore, where a change in disease occurs, we should be vigilant of the possibility of secondary fungal infection. Early and timely diagnosis will help improve the prognosis.

The study has several limitations that should be acknowledged. First, the relatively small sample size may restrict the generalizability and statistical significance of the findings. Additionally, data were sourced from specific regions or institutions, which might not fully represent the broader population. Furthermore, the methods and equipment used might have inherent limitations affecting the accuracy and reproducibility of the results. Not all potential confounding factors were controlled, which could influence the interpretation and applicability of the findings. Lastly, the study may not fully account for individual differences, such as genetic background or lifestyle factors, which could impact the results. Future research should address these limitations by increasing sample sizes, incorporating data from multiple centers, extending study durations, employing advanced methodologies, and better controlling for confounding variables. Additionally, a focus on individual differences could provide a more comprehensive understanding of the findings.

## Acknowledgments

The authors appreciate the patient’s son consent to present this case.

## Author contributions

**Conceptualization:** Yufang Wang.

**Data curation:** Jin Yu, Yufang Wang, Rong Xu.

**Formal analysis:** Junjun Wu.

**Investigation:** Junjun Wu, Jin Yu, Rong Xu.

**Methodology:** Junjun Wu.

**Project administration:** Yufang Wang.

**Resources:** Jin Yu.

**Supervision:** Huaming Li, Yufang Wang.

**Writing – original draft:** Junjun Wu.

**Writing – review & editing:** Huaming Li.
